# Antimicrobial resistance profiles of bacteria from clinical specimens at Amhara Public Health Institute, Bahir Dar, Ethiopia: A retrospective study

**DOI:** 10.1371/journal.pone.0337332

**Published:** 2025-12-05

**Authors:** Michael Getie, Wudu Tafere, Alem Tsega, Tsehaynesh Gebreyesus, Gizeaddis Belay, Alemayehu Abate, Hailu Getachew, Bayeh Abera, Demeke Endalamaw, Tazeb Molla, Teshiwal Deress, Belay Bezabih

**Affiliations:** 1 Medical Microbiology, Amhara Public Health Institute, Bahir Dar, Ethiopia; 2 Medical Biotechnology, Amhara Public Health Institute, Bahir Dar, Ethiopia; 3 Department of Medical Microbiology, College of Medicine and Health Sciences, Bahir Dar University, Bahir Dar, Ethiopia; 4 Department of Quality Assurance and Laboratory Management, School of Biomedical and Laboratory Sciences, College of Medicine and Health Sciences, University of Gondar, Ethiopia; 5 Field Epidemiology, Amhara Public Health Institute, Bahir Dar, Ethiopia; Debre Markos University, ETHIOPIA

## Abstract

**Background:**

Antimicrobial resistance is a major public health problem worldwide, particularly in developing countries. The effectiveness of currently available antimicrobial is decreasing due to the increasing prevalence of resistant strains among bacterial isolates. This study aims to determine the antimicrobial resistance profile of bacterial isolates from different clinical specimens at Amhara Public Health Institute.

**Materials and methods:**

A retrospective analysis was conducted using data extracted from the WHONET 2024 database from July 1, 2022, to December 31, 2024, at the Amhara Public Health Institute bacteriology and mycology reference laboratory. The age range of the patients included in this study was 1–96 years, and the mean age of the patients was 33.44 ± 17.36 years. The data included demographic characteristics of patients, types of bacterial isolates and antimicrobial resistance profiles, which were analyzed using SPSS version 20 statistical software. The descriptive statistics were displayed as percentages and frequencies. The chi-square test was used to determine the prevalence of bacterial isolates from patients by age and sex. P-values below 0.05 were seen as a sign of a statistically significant difference.

**Results:**

A total of 1165 specimens were processed, resulting in a culture-positive rate of 41% (478/1165) for bacterial pathogens isolated from clinical specimens. The majority of bacterial isolates were from stool (55%; 263/478), urine (20%; 96/478), wound (12.9%; 62/478), and blood (9.8%; 45/478), respectively. Of these, Gram-negative bacteria accounted for 89.1% (426/478) and Gram-positive bacteria accounted for 10.8% (52/478). The predominant bacterial isolates were *Vibrio cholerae* 54.6% (261/478), *E. coli* 16.1% (77/478), *Klebsiella* spp 6% (29/478), *S. aureus* 4.6% (22/478) and *Enterococcus* spp 2.9% (14/478). In this study *Proteus* spp 67.6% (46/68), *A. baumannii* 58.4% (31/53), and *Klebsiella* spp 64.1% (136/212) were identified as the most resistant bacteria to the tested antimicrobial. *S. aureus* shows resistance to tobramycin 100% (1) and penicillin 100% (17), oxacillin 84.6% (11/13) and tetracycline 63.6% (7/11). *Enterococcus* spp resistance to vancomycin 85.7% (6/7), penicillin 72.7% (8/11) and ampicillin 62.5% (5/8). In total, 53.1% (254/478) of the bacterial isolates were classified as multidrug-resistant (MDR), with 93.7% (238/ 254) being Gram-negative bacteria.

**Conclusions:**

Both Gram-negative and Gram-positive isolates showed high levels of resistance to commonly used antimicrobial. To address the problem of antimicrobial resistance, healthcare providers should focus on responsible antimicrobial prescribing practices based on local antibiogram data.

## Introduction

Antimicrobial resistance (AMR) is a phenomenon in which microorganisms become resistant to antimicrobial agents to which they were originally sensitive [1]. Antimicrobial resistance is a major Public health problem worldwide, particularly in developing countries where infectious diseases, poverty and malnutrition are endemic [2]. Recent data shows that about 700,000 death per year are attributable to AMR infections and projected to increase to 10 million annually by 2050 if the present trends persist [3].

Multidrug-resistant (MDR) bacteria are difficult to treat, limit therapeutic options, prolong hospitalization, require higher doses, and have higher tendencies for toxicity [4]. The slow progress in research and development of novel antimicrobial, due to the emergence of MDR pathogens [5].

The problem of antimicrobial resistance is not only the cause of the development of resistance but also the transmission of the resistant strains from one person to another, especially in a health facility setting [6]. The problem worsens in Ethiopia, due to multiple factors including lack of surveillance systems, limited resources, poor infection prevention and control practice, misuse and overuse of antimicrobial, and lack of clinical microbiology laboratories to identify the specific etiologic agents and their antimicrobial susceptibility testing has increased empirical therapy, which in turn leads to the emergence of AMR [7].

In Ethiopia, identifying the most common bacterial pathogens and their resistance patterns is crucial to optimizing therapy and ultimately reducing the morbidity and mortality linked to infectious diseases [8]. This study aimed to identify the bacterial pathogens and their antimicrobial resistance profiles in clinical specimens sent to the Amhara Public Health Institute (APHI).

## Materials and methods

### Study area

The study was conducted at Amhara Public Health Institute, Bahir Dar city, Amhara Regional State, Ethiopia which is approximately 565 km away from the capital city, Addis Ababa. The town has a latitude of 12^o^361 N and a longitude of 37^o^281 E with an elevation of 2133 meters above sea level. The institute provides healthcare services to over twenty-five million people in the region. It has an accredited reference level laboratory with 7 sections and a separate reception room. It was accredited by the Ethiopian National Accreditation Office. The microbiology section is one of the principal areas; it is estimated that 1,600 clinical specimens are delivered annually. This section provides accredited diagnostic laboratory testing services, including microscopy, culture, organism identification, and antimicrobial susceptibility testing (AST) for patients or specimens referred to from zonal and regional health facilities.

### Study design and period

An institution-based retrospective study was conducted by accessing the APHI bacteriology and mycology reference laboratory WHONET 2024 database from July 1, 2022, to December 31, 2024.

### Study population

The dataset comprised 1,165 patient specimens with suspected bacterial infections. Demographic characteristics patients and culture results were accessible in the APHI bacteriology and mycology reference laboratory WHONET 2024 database from July 1, 2022, to December 31, 2024.

### Sampling methods

The study used a comprehensive sampling method that incorporated all bacteriological culture records of patients of any age who were suspected of having a bacterial infection during the study period.

### Inclusion and exclusion criteria

All specimen entries on the WHONET 2024 database during the study period, having information on the age of a patient, sex, source of specimen, type of specimen, hospital units, isolated organism, and antimicrobial resistance profile, were included in this study. However, entries without any of the aforementioned information or specimens with unknown specimen type, unknown source of specimen, and specimens without culture result status were excluded from this study.

### Bacterial isolation and identification

The standard operating procedures (SOPs) of specimen collection and transportation of different clinical specimens were implemented. The collected clinical specimens were delivered to the bacteriology and mycology reference laboratory and processed following standard procedures. Conventional microbiological culture methods were employed to isolate and identify bacteria. Media was prepared in-house as per procedures stipulated in Cheesbrough [8]. Clinical specimens, including urine, blood, sputum, wound/pus, cerebrospinal fluid, body fluid, discharge (ear/eye), throat, and sputum, were cultured. Each clinical sample employed standard microbiological culturing techniques. Specimens were inoculated into the appropriate isolation culture media and incubated at 35–37 °C, according to standard protocols for each sample. Bacterial identification was made mainly based on colony characteristics, Gram stain reaction, and proper biochemical tests as per suitability according to CLSI guidelines [9] and developed SOPs. Identification of Gram-positive bacteria was done using Gram stain, hemolytic activity on sheep blood agar plates, catalase reaction, and coagulase test. Gram-negative bacteria were identified based on colony morphology on blood agar and MacConkey agar, followed by biochemical reactions, namely oxidase, triple sugar iron (TSI), sulphur indole and motility (SIM), citrate, lysine decarboxylase (LDC), and urease tests. After bacterial identification, antimicrobial susceptibility tests were done on Mueller-Hinton agar (Oxoid Basingstoke, UK) using the Kirby-Bauer disk diffusion method [10].

### Antimicrobial susceptibility testing

Antimicrobial susceptibility testing of the isolates was performed by the Kirby–Bauer disk diffusion test method on Mueller–Hinton agar for the following antimicrobial agents (Oxoid, Basingstoke, Hampshire, UK) [11,12]. Standard antimicrobial discs with specified concentrations were used to detect the resistance patterns of each isolate. The plates were incubated overnight. After incubation was completed, the zone inhibition diameter was measured in millimeters. The zones were interpreted as susceptible, intermediate, or resistant according to CLSI 2024 [9]. However, during antimicrobial susceptibility testing and reporting considerations for each organism group include agents of proven test efficacy that show acceptable in vitro test susceptibility and effective clinically be analyzed and reported as susceptible. The definition of CDC was used in this study for MDR: resistance of bacterial isolates to at least one antimicrobial in three or more drug classes [13]. The following standard antimicrobial, with abbreviated names and disk contents in brackets, were used to test the resistance profiles of bacterial isolates: Gram-positive isolates were tested for ampicillin (AMP) (10 µg), cefoxitin (FOX) (30 µg), clindamycin (DA) (2 μg), ciprofloxacin (CIP 5 μg), ceftriaxone (CRO) (30 µg), chloramphenicol (CHL) (30 µg), erythromycin (ERY) (15 μg), gentamicin (GEN) (10 µg), penicillin (PEN) (10 μg), nitrofurantoin (NIT) (300 μg), trimethoprim-sulphametazol (SXT) (1.25/23.75 µg), tetracycline (TCY) (30 μg), tobramycin (TOB) (10 µg) and vancomycin (Van) (30 µg) [9,14]. Gram-negative isolates were tested for ampicillin (AMP) (10 µg), amoxicillin-clavulanic acid (AMC) (20/10 µg), ceftazidime (CAZ) (30 µg), ceftriaxone (CRO) (30 µg), ciprofloxacin (CIP) (5 µg), chloramphenicol (CHL) (30 µg), gentamicin (GEN) (10 µg), meropenem (MEM) (10 µg), tobramycin (TOB) (10 µg), trimethoprim-sulphmetaxzole (SXT) (1.25/23.75 µg)and tetracycline (TCY) (15 µg) [9,14].

### Data source and access

Data for this study were obtained from the APHI bacteriology and mycology reference laboratory, WHONET 2024, a software database tool developed by the World Health Organization for antimicrobial resistance surveillance [15]. This electronic database included all clinical culture data collected from July 1, 2022, to December 31, 2024. The data was accessed on January 6, 2025, exclusively for this study. The data accessed was aggregated through Microsoft Excel 2013 and analyzed using SPSS version 20.

### Data quality control

Standard operating procedures for bacteriological techniques were followed throughout clinical specimen collection, transportation, culture media preparation, bacterial isolation, identification and antimicrobial susceptibility testing. Culture media sterility was ensured by random selection and incubation of 5% of prepared media. Media performance was regularly evaluated using known standard strains of *E. coli* (ATCC25922), *S. aureus* (ATCC25923) and *P. aeruginosa* (ATCC 27853). The WHONET 2024 database offers a number of quality control systems and alerts to check data quality.

### Data analysis

Data extracted from the WHONET 2024 database [16], aggregated through a Microsoft Excel 2013 spreadsheet for cleaning and validation, and then transferred to SPSS version 20 software for analysis. Descriptive statistics were used to designate the demographic characteristics of the participants, magnitudes of bacterial isolates and antimicrobial resistance profiles of the isolates. Chi-square test was employed to reveal age and sex specific prevalence of bacterial isolates from patients. P-value of less than 0.05 was considered to indicate statistically significant difference. Finally, the results of the findings were presented as frequency and percentage in texts, tables, and graphical forms.

### Ethics approval

This manuscript does not involve the use of data from any animal or bio specimens from deceased individuals. This retrospective study using WHONET 2024 database, the IRB fully waived the requirement for informed consent. We obtain permission from the relevant laboratory diagnostic directorate and APHI general director. The regional public health research ethical review committee granted ethical approval in May 2024 under the number NoH/R/T/T/D/07/74.

## Results

### Demographic characteristics of patients with bacterial isolates

A total of 1165 specimens were included in this study that met the eligibility criteria from different clinical specimens (stool, urine, blood, wound, genital/ urethral, discharge (ear/eye), and CSF. The mean age of the patients was 33.44 ± 17.36 years, and male patients accounted for 54.1% (631/1165). The age of the study participants ranged from 1 day to 96 years. Most of the study participants were between the ages of 15 and 64 years, 78.2% (912/1165). Most specimens 71.2% (829/1165) were from the medical outpatient department (MOPD), 25.7% (299/1165) from the emergency outpatient department (EOPD), 20.9% (244/1165) from the inpatient ward, and 1.1% (13/1165) specimens were from the community (Table 1).

**Table 1:** Patient profiles, clinical specimens, hospital unites, and culture results.

Variables		Frequency	Percent
Sex	Male	631	54.2
	Female	534	45.8
Age groups	0-4	104	8.9
	5 −14	78	6.7
	15-64	912	78.2
	65+	71	6.1
Hospital units	Medical OPD	829	71.2
	Emergency OPD	299	25.7
	Inpatient Ward	24	2.1
	Other	13	1.1
Clinical specimens	Stool	486	41.7
	Urine	369	31.7
	Blood	155	13.3
	Wound/pus	107	9.2
	Genital/Ureteral	16	1.4
	Discharge (ear/eye)	16	1.4
	Cerebrospinal fluid	9	0.8
	Body fluid	5	0.4
	Sputum	1	0.9
	Throat	1	0.9
Gram stain results	Gram-negative bacteria	426	88.4
	Gram-positive bacteria	52	10.8
	Candida albicans	4	0.8
	No growth	683	58.6

Other-community, OPD-out patient department.

Overall, 41% (478/1165) of the specimens were positive for aerobic bacterial isolates. Of these, 89.1% (426/478) were Gram-negative bacteria and 10.8% (52/478) were Gram-positive bacteria and the highest isolation rates were obtained from 15–64 years age group 77.2% (369/478) not statistically significant (P = 0.424).The isolated bacteria was relatively higher in males 56.1% (268/478) than females 43.9% (210/478) though not statistically significant (p = 0.26).The most frequently culture-processed specimens were stool 41.7% (486/1165) and urine 31.7% (369/1165) (Table 1 and Table 2).

**Table 2:** Age and sex specific prevalence of bacterial isolates from patients.

Variables	Frequency	Culture positives	Prevalence (%)	ᵪ2 (p. value)
Sex				1.183 (0.26)
Male	631	268	56.1	
Female	534	210	43.9	
**Age groups**				2.971 (0.424)
0-4	104	48	10	
5-14	78	28	5.9	
15-64	912	369	77.2	
65+	71	33	6.9	

ᵪ2- chi-square.

### The magnitudes of bacterial isolates

The most frequently isolated Gram-negative bacteria were *Vibrio cholerae* 54.6% (261/478) and *E. coli* 16.1% (77/478). *S. aureus* 4.6% (22/478) and *Enterococcus* spp 2.9% (14/478) were the predominant isolated Gram-positive bacteria. Most of the bacterial isolates were from stool specimens, 55.4% (265/478) followed by urine, 20.5% (98/478) and wound, 12.1% (58/478) (Table 3).

**Table 3:** The magnitudes of bacterial isolates from clinical specimen.

Bacterial isolates	Clinical specimens							Total
	Blood		Discharge (ear/eye)	Genital	Cerebrospinal fluid	Stool	Urine	Wound
*A. baumannii*	6 (1.2)	0	0	0	0	1(0.2)	3(0.6)	10 (2)
*Citrobacter* spp	0	0	0	0	0	4 (0.8)	2 (0.4)	6 (1.2)
*Klebsiella* spp	5 (1)	0	0	2 (0.4)	1 (0.2)	13 (2.7)	8(1.6)	29 (6)
*Enterobacter cloacae*	0	1 (0.2)	0	0	0	1 (0.2)	1 (0.2)	3 (0.6)
*E. coli*	3 (0.6)	1 (0.2)	0	1 (0.2)	1 (0.2)	52 (10.8)	19(3.9)	77(16.1)
*Enterococcus* spp	11 (2.3)	0	0	0	0	3 (0.6)	0	14 (2.9)
*Pseudomonas aeruginosa*	0	1 (0.2)	1 (0.2)	2 (0.4)	0	14 (2.9)	7(1.4)	25 (5.2)
*Proteus* spp	0	0	0	0	0	2 (0.4)	8 (1.6)	10 (2)
*Salmonella typhi*	0	0	0	0	2 (0.4)	0	0	2 (0.4)
*S. aureus*	5 (1)	2 (0.4)	0	0	0	6 (1.2)	9 (1.8)	22 (4.6)
*Vibrio cholera*	0	0	0	0	261 (54.6)	0	0	261 .(54.6)
*Streptococcus viridians*	5 (1)	0	0	0	0	0	0	5 (1)
*Others*	12 (2.5)	0	1 (0.2)	0	0	0	1 (0.2)	14 (2.9)
Total	47 (9.8)	5 (1)	2 (0.4)	5 (1)	265 (55.4)	96 (20)	58 (12.1)	478 (100)

Key: Others include Coagulase-negative staphylococci, *Providencia* spp, *S.pyogenes*, *Haemophilus* spp and *Serratia* spp.

### Antimicrobial resistance in Gram-negative bacteria

**Fig 1:**
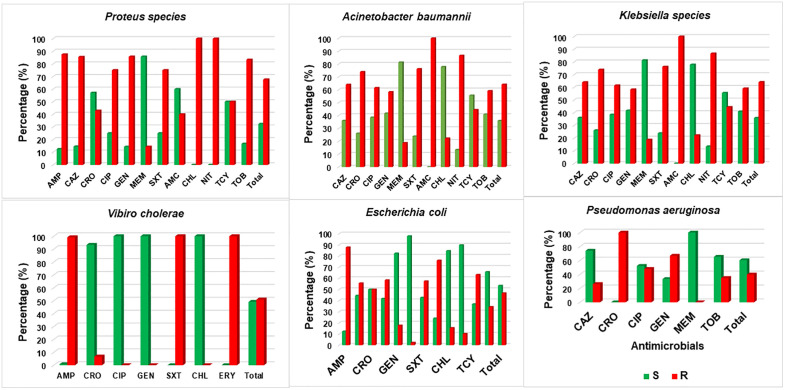
Antimicrobial resistance levels of the six major top isolated Gram-negative bacteria.

In this study, *Proteus* spp 67.6% (46/68), *A. baumannii* 58.4% (31/53), and *Klebsiella* spp 64.1% (136/212) were identified as the most resistant bacteria to the commonly used antimicrobial. These bacteria exhibited resistance to ceftazidime, ciprofloxacin, and trimethoprim-sulfamethoxazole. Specifically, *Proteus* spp showed 85.7% (6/7) resistance to ceftazidime, 75% (6/8) to ciprofloxacin, and 75% (6/8) to trimethoprim-sulfamethoxazole. *A. baumannii* resistance rate of 77.8% (7/9) to ceftazidime, 62.5% (5/8) ciprofloxacin and 40% (2/5) to trimethoprim-sulfamethoxazole. *Klebsiella* spp displayed 64% (16/25) resistance to ceftazidime, 61.5% (5/8) to ciprofloxacin, and 76.2% (16/21) to trimethoprim-sulfamethoxazole. Furthermore, *E. coli*, *Proteus* spp, and *Vibrio cholerae* were resistant to ampicillin, 87.7% (57/65), 87.5% (6/7) and 99.2% (122/123), respectively (Fig 1).

Antimicrobial resistance (AMR) profile of Gram-negative bacteria showed that erythromycin 99.1% (116/117), ampicillin 94% (188/200), trimethoprim-sulfamethoxazole 82.1% (188/229), amoxicillin-clavulanic acid 81.5% (66/81), ceftazidime 54.7% (80/146) and tetracycline 51.5% (17/33) was substantially resisted antimicrobial (Table 4).

**Table 4:** Antimicrobial resistance profile of Gram-negative bacterial isolates.

Bacteria		AMP	CAZ	CRO	CIP	GEN	MEM	SXT	AMC	CHL	NIT	TCY	TOB	ERY	Total
A.baumannii	#T	NA	9	5	8	10	8	5	NA	NA	NA	NT	8	NA	53
	R (%)	NA	7 (77.8)	1 (20)	5 (62.5)	7 (70.0)	3 (37.5)	2 (40)	NA	NA	NA	NT	6 (75)	NA	31 (58.4)
Citrobacter spp	#T	NA	4	3	4	4	4	3	NT	2	2	NT	4	NA	30
	R (%)	NA	4 (100)	-	3 (75)	2 (50)	-	2 (66.7)	NT	1 (50)	2(100)	NT	2 (50)	NA	16 (53.3)
Klebsiella spp	#T	NA	25	23	26	24	16	21	22	9	15	9	22	NA	212
	R (%)	NA	16 (64)	17(73.9)	16 (61.5)	14 (58.3)	3 (18.7)	16 (76.2)	22 (100)	2 (22.2)	13 (86.6)	4 (44.4)	13 (59.1)	NA	136 (64.1)
Enterobacter cloacae	#T	NA	3	3	3	3	2	2	2	1	1	2	2	NA	25
	R (%)	NA	1 (33.3)	2 (66.6)	1 (33.3)	1 (33.3)	1 (50)	1 (50)	2 (100)	1(100)	1 (100)	-	2 (100)	NA	13 (52)
E. coli	#T	65	72	64	72	62	45	61	50	13	49	19	67	NA	639
	R (%)	57 (87.7)	40 (55.6)	32 (50)	42 (58.3)	11(17.7)	1 (2.2)	35 (57.4)	38 (76)	2 (15.4)	5 (10.2)	12(63.2)	23 (34.3)	NA	298 (46.6)
P.aeruginosa	#T	NA	23	2	23	6	1	NA	NA	NA	NA	NA	23	NA	78
	R (%)	NA	6 (26.1)	2 (100)	11(47.8)	4 (66.7)	-	NA	NA	NA	NA	NA	8 (34.8)	NA	31 (39.7)
Proteus spp	#T	8	7	7	8	7	7	8	5	2	1	2	6	NA	68
	R (%)	7 (87.5)	6 (85.7)	3 (42.9)	6 (75.0)	6 (85.7)	1 (14.3)	6 (75.0)	2 (40)	2(100)	1(100)	1 (50)	5 (83.3)	NA	46 (67.6)
Salmonella typhi	#T	2	NT	NT	2	NA	NT	2	NT	NT	NA	NT	NA	NA	6
	R (%)	2 (100.0)	NT	NT	0 (0.0)	NA	NT	1 (50.0)	NT	NT	NA	NT	NA	NA	3 (50.0)
Vibrio cholerae	#T	123	NT	119	120	1	NT	123	NT	122	NT	NT	NT	116	724
	R (%)	122(99.2)	NT	8 (6.7)	0 (0.0)	0 (0.0)	NT	123 (100)	NT	-	NT	NT	NT	116 (100)	369 (50.9)
Others	#T	2	3	1	3	3	1	3	2	2	NT	1	2	1	28
	R (%)	-	-	-	2 (66.6)	1 (33.3)	-	2(66.6)	2(100)	-	NT	-	-	-	7 (25)
Total	#T	200	146	228	272	120	84	229	81	149	68	33	134	117	1863
	R (%)	188 (94)	80 (54.7)	65 (28.5)	86 (31.6)	46 (38.3)	9 (10.7)	188 (82.1)	66 (81.5)	8 (5.4)	22 (32.3)	17 (51.5)	59 (44.0)	116 (99.1)	950 (50.9)

AMP-ampicillin, CAZ-ceftazidime, CRO-ceftriaxone, CIP-ciprofloxacin, GEN-gentamicin, MEM-meropenem, SXT- trimethoprim-sulfamethoxazole, AMC-amoxicillin-clavulanic acid, CHL-chloramphenicol, NIT-nitrofurantoin, TCY- tetracycline, TOB-tobramycin, ERY-erythromycin, NA-not analyzed (during antimicrobial susceptibility testing and reporting antimicrobial were selected based on CLSI, 2024 for each organism group but agents of proven test efficacy that show unacceptable in vitro test susceptibility and ineffective clinically should not be analyzed), NT- not tested (antimicrobial were not tested due unavailability of antimicrobial agents in bacteriology and mycology reference laboratory).

### Antimicrobial resistance profiles of Gram-positive bacteria

**Fig 2:**
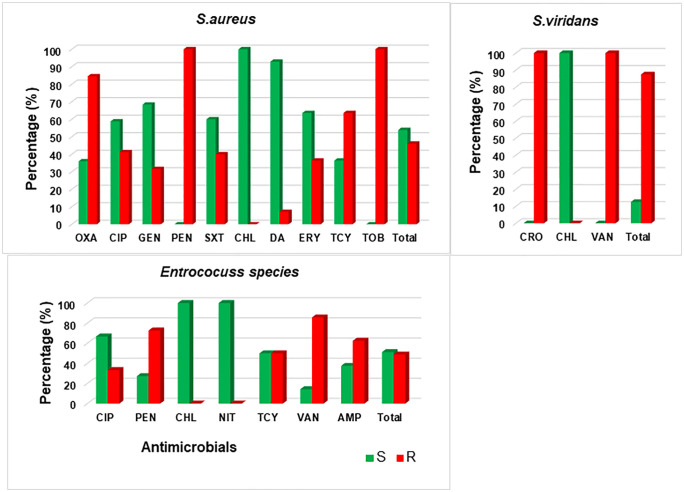
Antimicrobial resistance levels of the three major top isolated Gram-positive bacteria.

Gram-positive bacteria showed resistance to tobramycin 100% (1), vancomycin 91.6% (11/12), oxacillin 87.5% (14/16), penicillin 83.3% (25/30), ceftriaxone 66.6% (2/3) and tetracycline 60% (9/15). But remained highly susceptible to chloramphenicol 100% (5), nitrofurantoin 100% (22) and clindamycin 93.7% (15/16). Among Gram-positive bacteria, *S. aureus* resistance to tobramycin 100% (1), penicillin 100% (17), oxacillin 84.6% (11/13), and tetracycline 63.6% (7/11). *Enterococcus* spp exhibited resistance to vancomycin 85.7% (6/7), penicillin 72.7% (8/11) and ampicillin 62.5% (5/8). *S. viridians* were resistant to ceftriaxone 100% (2) and vancomycin 100% (5) seen in (Fig 2 and Table 5).

**Table 5:** Antimicrobial resistance profile of Gram-positive bacteria isolates.

Antimicrobial	*Enterococcus* spp		*S. aureus*		CONS		*S. pyogenes*		*S. viridians*		Total	
	#T	R (%)	#T	R (%)	#T	R (%)	#T	R (%)	#T	R (%)	#T	R (%)
OXA	NA	NA	13	11 (84.6)	3	3 (100)	NA	NA	NA	NA	16	14 (87.5)
CRO	NA	NA	1	0 (0.0)	NA	NA	NT	NT	2	2 (100.0)	3	2 (66.6)
CIP	3	1 (33.3)	17	7 (41.2)	3	2 (66.7)	NA	NA	NA	NA	23	10 (43.5)
GEN	NT	NT	19	6 (31.6)	4	2 (50.0)	NA	NA	NA	NA	23	8 (34.8)
PEN	11	8 (72.7)	17	17 (100)	1	0 (0.0)	1	0 (0.0)	NA	NA	30	25 (83.3)
SXT	NA	NA	15	6 (40)	2	2 (100)	NA	NA	NA	NA	17	8 (47.0)
CHL	11	0 (0.0)	7	0 (0.0)	3	0 (0.0)	NT	NT	1	0 (0.0)	22	0 (0.0)
DA	NA	NA	14	1 (7.1)	2	0 (0.0)	NT	NT	NT	NT	16	1 (6.3)
ERY	NA	NA	11	4 (36.4)	NT	NT	1	0 (0.0)	NT	NT	12	4 (33.3)
NIT	1	0 (0.0)	4	0 (0.0)	NT	NT	NA	NA	NA	NA	5	0 (0.0)
TCY	2	1 (50.0)	11	7 (63.6)	1	1 (100)	1	0 (0.0)	NA	NA	15	9 (60.0)
TOB	NA	NA	1	1 (100)	NT	NT	NA	NA	NA	NA	1	1 (100.0)
AMP	8	5 (62.5)	NA	NA	NA	NA	1	0 (0.0)	NA	NA	9	5 (55.5)
VAN	7	6 (85.7)	NA	NA	NA	NA	NT	NT	5	5(100.0)	12	11 (91.6)
Total	43	21 (48.8)	130	60 (46.1)	19	10 (52.6)	4	0 (0.0)	8	7 (87.5)	204	98 (48)

OXA- oxacillin, CRO- ceftriaxone, CIP-ciprofloxacin, GEN- gentamicin, PEN- penicillin, SXT- trimethoprim-sulfamethoxazole, CHL-chloramphenicol, DA- clindamycin, ERY-erythromycin, NIT-nitrofurantoin, TCY- tetracycline, TOB-tobramycin, AMP- ampicillin, VAN-vancomycin, NA-not analyzed (during antimicrobial susceptibility testing and reporting antimicrobial were selected based on CLSI, 2024 for each organism group but agents of proven test efficacy that show unacceptable in vitro test susceptibility and ineffective clinically should not be analyzed), NT- not tested (antimicrobial were not tested due unavailability of antimicrobial agents in bacteriology and mycology reference laboratory).

### Multidrug resistance profiles of bacterial isolates

In a total of 478 bacterial isolates, 53.1% (254) were identified as multidrug resistant (MDR). A significant proportion of these, 93.7% (238/254), were Gram-negative bacteria, while 6.3% (16/254) were Gram-positive bacteria. Among the MDR Gram-negative bacteria, *Proteus* spp, *A. baumannii*, *Klebsiella* spp and *E. coli* showed highest MDR rates of 90% (9/10), 80% (8/10), 79.3% (23/29) and 79.2% (61/77) respectively (Table 6).

**Table 6:** Multidrug resistance profiles of bacterial isolates.

Bacterial isolates	Degree of resistance									
	R0 n (%)	R1 n (%)	R2 n (%)	R3 n (%)	R4 n (%)	R5 n (%)	R6 n (%)	R7 n (%)	R 8 n (%)	MDR* n (%)
*Enterococcus* spp (n = 14)	1 (7.1)	5 (35.7)	6 (42.9)	2 (14.2)	0	0	0	0	0	2 (14.2)
*S. aureus* (n = 22)	3 (13.6)	4 (18.1)	3 (13.6)	6 (27.3)	3 (13.6)	0	3 (13.6)	0	0	12 (54.5)
CONS (n = 10)	7 (70)	0	1 (10)	2 (20)	0	0	0	0	0	2 (20)
*S. pyogenes* (n = 1)	0	1 (100.0)	0 (0.0)	0	0	0	0	0	0	0 (0.0)
*S. viridians* (n = 5)	0	3 (60.0)	2 (40.0)	0	0	0	0	0	0	0 (0.0)
*A. baumannii* (n = 10)	0	2 (20.0)	0	1 (10)	3 (30)	4 (40)	0	0	0	8 (80)
*Citrobacter* spp (n = 6)	2 (33.3)	0	0	1 (16.6)	2 (33.3)	1 (16.6)	0	0	0	4 (66.6)
*Klebsiella* spp(n = 29)	1(3.4)	2 (6.8)	2 (6.8)	5 (17.2)	2 (6.8)	5 (17.2)	7 (24.1)	3 (10.3)	1 (3.4)	23 (79.3)
*Enterobacter cloacae* (n = 3)	0	1 (33.3)	0 (0.0)	0	0	1 (33.3)	1 (33.3)	0	0	2 (66.6)
*E. coli* (n = 77)	2 (2.6)	7 (9.1)	7 (9.1)	8 (10.4)	13 (16.9)	9 (69.2)	11 (14.3)	13 (16.9)	7 (9.1)	61 (79.2)
*P. aeruginosa* (n = 25)	7 (28)	5 (20)	4 (16)	6 (24)	3 (12)	0	0	0	0	9 (36)
*Proteus* spp (n = 10)	1 (10)	0 (0.0)	0 (0.0)	1(10)	1(10)	1(10)	2 (20)	1(10)	3 (30)	9 (90)
*Salmonella typhi* (n = 2)	0	1 (50.0)	1 (50.0)	0	0	0	0	0	0	0
*Vibrio cholerae* (n = 261)	139(52.8)	0	0	113 (43.3)	9 (13.1)	0	0	0	0	122 (46.7)
Others (n = 3)	0	0	3 (100)	0	0	0	0	0	0	0
Total (n = 478)	163 (34.1)	31 (6.5)	29 (6.1)	145 (30.3)	36 (7.5)	21 (4.4)	24 (5.1)	17 (3.6)	11 (2.3)	254 (53.1)

*MDR – Isolates resistant to 3 or more antimicrobial classes, R0- no antimicrobial resistant, R1 - resistant to one antimicrobial classes, R2 - resistant to two antimicrobial classes, R3- resistant to three antimicrobial classes, R4- resistant to four antimicrobial classes, R5-resistant to five antimicrobial classes, R6- resistant to six antimicrobial classes, R7 - resistant to seven antimicrobial classes, R8- resistant to eight antimicrobial classes.

## Discussion

Antimicrobial resistance (AMR) is a critical global health challenge, threatening our ability to effectively treat infections and manage complications in health care facilities. In the current study, overall prevalence of culture-confirmed bacterial isolates was 41% (478/1165) from clinical specimens. This frequency is comparable to a study in Debre Markos (48.7%) [17]. However, the current study’s rate was higher than findings from Southern Ethiopia (32.6%) [18], Addis Ababa (32.8%) [19], Gondar (21.6%, 14.8%) [20,21], Jimma (22.1%) [22], Ghana (37.8%) [23], Nigeria (29.6%) [24], and Yemen (43.2%) [24] respectively. However, this was lower than a study conducted in Bahir Dar (61.6%) [25], Gondar (83.9%) [26] and India (91.3%) [27]. The most possible explanation could be due to the difference in culture identification technique in the study population, the study design, geographical location, etiological agents, and infection prevention and control policies between regions and countries [2,13,24].

In this study higher proportion of Gram-negative bacteria 89.1% (426/478) compared to Gram-positive bacteria 10.8% (52/478) were isolated. Similarly, studies reported in Ethiopia (52.1% vs 47.9%) [28], (71.2% vs 28.8%) [29], (62.8% vs 37.2%) [30], (69% vs. 31%) [31], (57.6% vs 39.4% vs 3.0% [32], Tehran (72.2% vs. 27.8%) [33], Egypt (57.5% vs 31.1%) [34], India (68.1% vs 31.9%) [27] and Iran (55%) vs 45%) [35] revealed that the predominant isolates were Gram-negative bacteria. This might be due to differences in their cell wall structure, the presence of an outer membrane protein and the specific types of bacteria prevalent in the study population. However, another study reported from Ethiopia (87.7% vs 12.3%) [36] and (77.4% vs 22.6%) [37], India (53.0% vs. 39.0%) [38] and Tehran (64.2% vs 33.5%) [33] showed higher bacterial isolates caused by Gram-positive bacteria than Gram-negative bacteria respectively. The dominance of Gram-positive bacterial isolates over Gram-negative isolates in might be due to specific environment which the bacteria were isolated, the types of infections [20].

This study revealed that *Vibrio cholerae* 54.6% (261/478), *E. coli* 16.1% (77/478) and *S. aureus* 4.6% (22/478) were the most prevalent isolates. This is in line with a study conducted in Ethiopia, *S. aureus* was the predominant isolate (31.5%) followed by *E. coli* (13.8%) [17], Iran *E.coli* (7.58%), *Vibrio cholerae* (66%)) [39], India *E coli* (12.8%) and *S. aureus* (8.4%)) [27], Nepal (*S. aureus*, *E. coli* and *Vibrio cholerae*) were 68%, 53% and 6% [40], respectively. In addition, *E. coli* (9%) and *S. aureus* (44%) were isolated from Malaysia [41]. This might be due to conflict and civil unrest, refugees under poor conditions and fragile health infrastructure [42].

A high percentage of Gram-positive bacteria exhibit resistance to multiple antimicrobials, including tobramycin 100% (1), vancomycin 91.6% (11/12), oxacillin 87.5% (14/16), penicillin 83.3% (25/30), ceftriaxone 66.6% (2/3), and tetracycline 60% (9/15). Similarly, high rates of resistant Gram-positive bacterial isolates reported in Debre Markos penicillin (89.7%) and tetracycline (71.3%) [17], Bahir Dar 65.4%, 42.6% and 34.6% were resistant to penicillin, tetracycline and oxacillin [43], Addis Ababa, penicillin (83.5%) and tetracycline (76.5%) [19], Egypt penicillin (89.5%) and oxacillin (76.52%) [20], Ruanda oxacillin (82.0%), penicillin (88%) and tetracycline (62%) [44], Malawi most bacteria exhibited high resistance to all commonly used antimicrobial excluding ciprofloxacin [45] and Nigeria penicillin (100%) [46] respectively. This concerning resistance is likely due to irrational use of antimicrobial and a lack of proper antimicrobial susceptibility testing in the region.

In the present study, Gram-negative isolates resistance to erythromycin 99.1% (116/117), ampicillin 94% (188/200), trimethoprim-sulfamethoxazole 82.1% (188/229), amoxicillin-clavulanic acid 81.5% (66/81) and ceftazidime 54.7% (80/146). This is in line with a study conducted in Debre Markos resistant to trimethoprim-sulfamethoxazole (53.1%) and ampicillin (70.4%) [17], Ceftazidime (77.2%) [36], Bahir Dar amoxicillin-clavulanic acid (90%) and ampicillin (85.7%) [43] and Gondar ceftriaxone (79.0%), trimethoprim-sulfamethoxazole(80.6%), amoxicillin-clavulanic acid (79.0%) were resistance [47].

Among Gram-negative bacterial isolates, *Klebsiella* spp resistance to ceftazidime 64% (16/25), ciprofloxacin 61.5% (5/8) and trimethoprim-sulfamethoxazole 76.2% (16/21). This is in line with a study conducted in Ethiopia resistant to ceftazidime (45%), ciprofloxacin (40%) and trimethoprim-sulfamethoxazole (45%) [48], trimethoprim-sulfamethoxazole (66.91%) [49], trimethoprim-sulfamethoxazole (100%) [50], trimethoprim-sulfamethoxazole (100%), ceftazidime (100%) and ciprofloxacin (90.9%) [51], ceftriaxone 43.3%) [52], ceftriaxone, trimethoprim-sulfamethoxazole and tetracycline with a pooled resistance range of 40.6–55.3%) [52]. A study in Sudan showed that resistance to ceftazidime (95.4%) [53], Iraq ceftazidime with a resistance rate of 100% [54], Bangladesh (ciprofloxacin and trimethoprim-sulfamethoxazole) was 40% and 45% [48] and South Africa trimethoprim-sulfamethoxazole (50%) [55]. These findings indicate a serious challenge in treating infections caused by these bacteria, as many commonly used antimicrobials are not effective.

In this study, *Proteus* spp showed 85.5% (6/7) resistance to ceftazidime, 75% (6/8) to ciprofloxacin, and 75% (6/8) to trimethoprim-sulfamethoxazole. This is line with a study conducted in Debre Berhan ceftazidime (99%) [56], Gondar ceftazidime (46.7%) [3], Nekemte ceftazidime (100%) [57], Egypt trimethoprim/sulfamethoxazole (80.6%), amoxicillin-clavulanic (57.3%) and ceftazidime (55.3%) [58], Congo ciprofloxacin (78.6%) and trimethoprim-sulfamethoxazole (100%) [59] and Sierra Leone ciprofloxacin (50%) [60]. But on the contrary, studies done at Nekemte, none of the isolates were resistant to ciprofloxacin [57]. The World Health Organization (WHO) categorizes antibiotic-resistant Gram-negative bacteria, including *Proteus* spp, high-priority pathogens due to their significant threat to public health. These bacteria are often resistant to last-resort antibiotics like carbapenems and third-generation cephalosporins, leading to increased mortality rates [61].

In total, 53.1% (254/478) of the bacterial isolates were classified as multidrug-resistant (MDR). This finding is consistent with study findings reported from Ethiopia (64.2%) [62], (56%) [63], (88.8%) [64], 70% [18], (78.57%) [65], 78.2% [66], 77.9% [67], and (77%) [68]. A comparable result was reported in the studies conducted in India 50% and 66.1% [69,70], China (42.5%) [71], Tanzania (70.5%) [72], Sierra Leone (64.3%) [73], Ghana (89.5%) [74], and Egypt (65.5%) [75]. However, the current study finding was higher than the study conducted in India (37.1%) [76], Nepal (42.6%) [77], Australia (36%) [78], Indonesia (28.7%) [79], the USA (27%) [80], France (11.6%) [81] and Tanzania (43.0%) [82]. This difference might be due to many factors, including sample size, sites of infection, study area, infection prevention practices and improper use of antimicrobial [83,84].

## Limitations of the study

The limitation of this study was that bacterial species were identified by phenotypic methods. Due to the retrospective nature of the data, we did not investigate risk factors for bacterial infection and antimicrobial resistance profiles.

## Conclusions

This study highlights a significant prevalence of antimicrobial resistance among bacterial isolates in the specified area, with a notable 41.4% (478/1165) of specimens yielding positive cultures. Higher rates of resistance to the commonly used antimicrobial agents were noticed for both Gram-negative and Gram-positive bacterial isolates. Moreover, MDR has been indicated in more than half of the bacterial isolates. Among bacterial isolates, a significant proportion 93.7% (238/254) of MDR were Gram-negative bacteria, which underscores the urgency of the situation. To effectively combat the issue of antimicrobial resistance, healthcare providers should prioritize judicious antibiotic prescribing practices, informed by local antibiogram data.

## Supporting information

S1 File Excel raw data. (XLSX)

## Abbreviations

AMR: Antimicrobial Resistance
APHI: Amhara Public Health Institute
ATCC: American type culture collection
CLSI: Clinical and laboratory standard Institute
CoNS: Coagulase-negative staphylococcus
CSF: Cerebrospinal fluid
ESBL: Extended spectrum ß-lactamase
IRB: Institutional review board
MDR: Multi drug resistance
SOP: Standard operating procedure
SPSS: Statistical package for social sciences
WHO: World Health Organization

## References

[1] Coculescu B-I. Antimicrobial resistance induced by genetic changes. J Med Life. 2009;2(2):114–23. 20108530 PMC3018982

[2] Organization WH. Antimicrobial resistance and primary health care. World Health Organization. 2018.

[3] Bitew G, Dagnew M, Dereje M, Birhanu A, Gashaw Y, Ambachew A, et al. Burden of multi-drug resistant bacterial isolates and its associated risk factors among UTI-confirmed geriatrics in Gondar town. Sci Rep. 2025;15(1):14270. doi: 10.1038/s41598-025-89123-9 40274838 PMC12022030

[4] Marino A, et al. The global burden of multidrug-resistant bacteria. Epidemiologia. 2025;6(2):21. 40407562 10.3390/epidemiologia6020021 PMC12101290

[5] Aslam B, Wang W, Arshad MI, Khurshid M, Muzammil S, Rasool MH, et al. Antibiotic resistance: a rundown of a global crisis. Infect Drug Resist. 2018;11:1645–58. doi: 10.2147/IDR.S173867 30349322 PMC6188119

[6] Halawa EM, Fadel M, Al-Rabia MW, Behairy A, Nouh NA, Abdo M, et al. Antibiotic action and resistance: updated review of mechanisms, spread, influencing factors, and alternative approaches for combating resistance. Front Pharmacol. 2024;14:1305294. doi: 10.3389/fphar.2023.1305294 38283841 PMC10820715

[7] Sulis G, Sayood S, Gandra S. Antimicrobial resistance in low- and middle-income countries: current status and future directions. Expert Rev Anti Infect Ther. 2022;20(2):147–60. doi: 10.1080/14787210.2021.1951705 34225545

[8] Cheesbrough M. District laboratory practice in tropical countries: Part 2. 2006.

[9] Lewis I, James S. Performance standards for antimicrobial susceptibility testing. 2024.

[10] Kibret M, Abera B. Antimicrobial susceptibility patterns of E. coli from clinical sources in northeast Ethiopia. Afr Health Sci. 2011;11 Suppl 1(Suppl 1):S40-5. doi: 10.4314/ahs.v11i3.70069 22135643 PMC3220125

[11] Cheesbrough M. District laboratory practice in tropical countries, part 2. Cambridge University Press. 2005.

[12] Hudzicki J. Kirby-Bauer disk diffusion susceptibility test protocol. American Society for Microbiology. 2009;15(1):1–23.

[13] Control C f. D, Prevention N center for emerging and zoonotic infectious diseases (NCEZID). One Health. 2017.

[14] Weinstein M. Clinical and laboratory standards institute (CLSI) M100-S25 document. Wayne, PA, USA: Clinical and Laboratory Standards Institute (CLSI). 2020.

[15] O’Brien TF, Eskildsen MA, Stelling JM. Using internet discussion of antimicrobial susceptibility databases for continuous quality improvement of the testing and management of antimicrobial resistance. Clin Infect Dis. 2001;33 Suppl 3:S118-23. 10.1086/321836 11524707 11524707

[16] Organization, W.H., WHONET 5: microbiology laboratory database software, in WHONET 5: microbiology laboratory database software. 1999. 10.1272/jnms.71.345 15514454

[17] Mulu W, Abera B, Yimer M, Hailu T, Ayele H, Abate D. Bacterial agents and antibiotic resistance profiles of infections from different sites that occurred among patients at Debre Markos Referral Hospital, Ethiopia: a cross-sectional study. BMC Res Notes. 2017;10(1):254. doi: 10.1186/s13104-017-2584-y 28683780 PMC5501330

[18] Hailemariam M, Alemayehu T, Tadesse B, Nigussie N, Agegnehu A, Habtemariam T, et al. Major bacterial isolate and antibiotic resistance from routine clinical samples in Southern Ethiopia. Sci Rep. 2021;11(1):19710. doi: 10.1038/s41598-021-99272-2 34611232 PMC8492677

[19] Kitila KT. Assessment of bacterial profile and antimicrobial resistance pattern of bacterial isolates from blood culture in Addis Ababa regional laboratory, Addis Ababa, Ethiopia. Clin Microbiol. 2018;7(312):2.

[20] Amsalu G, Moges F, Bayu G, Gelaw B. Magnitude and antimicrobial susceptibility profile of bacteria isolated from pediatric sepsis cases at University of Gondar Hospital, Northwest Ethiopia. BMC Pediatr. 2024;24(1):491. doi: 10.1186/s12887-024-04969-8 39090628 PMC11293073

[21] Deress T, Belay G, Ayenew G, Ferede W, Worku M, Feleke T, et al. Bacterial etiology and antimicrobial resistance in bloodstream infections at the University of Gondar Comprehensive Specialized Hospital: a cross-sectional study. Front Microbiol. 2025;16:1518051. doi: 10.3389/fmicb.2025.1518051 40182289 PMC11966405

[22] Kiya GT, Mekonnen Z, Asefa ET, Gudina EK, Ahmed H, Beyene G, et al. Bacterial isolates and antibiotic resistance in critically ill sepsis patients at a tertiary hospital in Ethiopia. BMC Infect Dis. 2025;25(1):1046. doi: 10.1186/s12879-025-11474-5 40830847 PMC12366403

[23] Dayie NT. Multidrug-Resistant Bacteria in Aquaculture Systems in Accra, Ghana. Environmental Health Insights. 2024;18:11786302241299369. 39600552 10.1177/11786302241299369 PMC11590155

[24] Chukwu EE, Abuh D, Idigbe IE, Omoregha P, Okwuraiwe AP, Ishola O, et al. Prevalence and associated risk factors of bacterial vaginosis among women of reproductive age living with, and without HIV in Lagos, Nigeria. BMC Womens Health. 2025;25(1):460. doi: 10.1186/s12905-025-04024-3 41023982 PMC12482457

[25] Hailu D. Drug resistance patterns of bacterial isolates from infected wounds at Bahir Dar regional health research laboratory center, Northwest Ethiopia. Ethiopian Journal of Health Development. 2016;30(3):112–7.

[26] Mohammed A, Seid ME, Gebrecherkos T, Tiruneh M, Moges F. Bacterial Isolates and Their Antimicrobial Susceptibility Patterns of Wound Infections among Inpatients and Outpatients Attending the University of Gondar Referral Hospital, Northwest Ethiopia. Int J Microbiol. 2017;2017:8953829. doi: 10.1155/2017/8953829 28386280 PMC5366191

[27] Jain P, Galiya A, Luke Philip S, Mateti UV, P S S, Gudi SK, et al. Bacteriological profile and antimicrobial resistance pattern among patients with sepsis: A retrospective cohort study. Int J Clin Pract. 2021;75(10):e14701. doi: 10.1111/ijcp.14701 34351692

[28] Mulu W, Abera B, Yimer M, Hailu T, Ayele H, Abate D. Bacterial agents and antibiotic resistance profiles of infections from different sites that occurred among patients at Debre Markos Referral Hospital, Ethiopia: a cross-sectional study. BMC Res Notes. 2017;10(1):254. doi: 10.1186/s13104-017-2584-y 28683780 PMC5501330

[29] Azimi T. Evaluating the antimicrobial resistance patterns among major bacterial pathogens isolated from clinical specimens taken from patients in Mofid Children’s Hospital, Tehran, Iran: 2013–2018. Infection and Drug Resistance, 2019: p. 2089–102. 10.2147/IDR.S215329 PMC6645606 31410032

[30] Alemayehu T, Ali M, Mitiku E, Hailemariam M. The burden of antimicrobial resistance at tertiary care hospital, southern Ethiopia: a three years’ retrospective study. BMC Infect Dis. 2019;19(1):585. doi: 10.1186/s12879-019-4210-1 31277588 PMC6612117

[31] Dagnew M, Yismaw G, Gizachew M, Gadisa A, Abebe T, Tadesse T, et al. Bacterial profile and antimicrobial susceptibility pattern in septicemia suspected patients attending Gondar University Hospital, Northwest Ethiopia. BMC Res Notes. 2013;6:283. doi: 10.1186/1756-0500-6-283 23875886 PMC3723433

[32] Tufa TB, Mackenzie CR, Orth HM, Wienemann T, Nordmann T, Abdissa S, et al. Prevalence and characterization of antimicrobial resistance among gram-negative bacteria isolated from febrile hospitalized patients in central Ethiopia. Antimicrob Resist Infect Control. 2022;11(1):8. doi: 10.1186/s13756-022-01053-7 35033191 PMC8761287

[33] Taki E. Microbial profile and antibiotic susceptibility pattern in diabetic patients with mild, moderate, and severe foot infections in Tehran. Archives of Razi Institute. 2022;77(5):1925. 37123144 10.22092/ARI.2022.359759.2476 PMC10133604

[34] Elghanam M, Emara M, Abdelhalim M, Moustafa W. Prevalence and Antibiotic Resistance Patterns of Multidrug-Resistant (MDR) Bacteria Isolated from Pediatric Intensive Care Units. Egyptian Journal of Medical Microbiology. 2024;33(1):0–0. doi: 10.21608/ejmm.2024.332173

[35] Azimi T, Maham S, Fallah F, Azimi L, Gholinejad Z. Evaluating the antimicrobial resistance patterns among major bacterial pathogens isolated from clinical specimens taken from patients in Mofid Children’s Hospital, Tehran, Iran: 2013-2018. Infect Drug Resist. 2019;12:2089–102. doi: 10.2147/IDR.S215329 31410032 PMC6645606

[36] Abebe T, Teklemariam Z, Shume T, Mekuria S, Urgesa K, Weldegebreal F. Bacterial Profile of External Ocular Infections, Its Associated Factors, and Antimicrobial Susceptibility Pattern among Patients Attending Karamara Hospital, Jigjiga, Eastern Ethiopia. Int J Microbiol. 2023;2023:8961755. doi: 10.1155/2023/8961755 36937542 PMC10023229

[37] Kitila K. Assessment of bacterial profile and antimicrobial resistance pattern. 2018.

[38] Gill MK, Sharma S. Bacteriological profile and antibiotic resistance pattern in blood stream infection in critical care units of a tertiary care hospital in North India. Ind Jour of Microb Res. 2016;3(3):270. doi: 10.5958/2394-5478.2016.00059.5

[39] Momtaz H, Dehkordi FS, Rahimi E, Asgarifar A. Detection of Escherichia coli, Salmonella species, and Vibrio cholerae in tap water and bottled drinking water in Isfahan, Iran. BMC Public Health. 2013;13:556. doi: 10.1186/1471-2458-13-556 23742181 PMC3703282

[40] Bantawa K, Sah SN, Subba Limbu D, Subba P, Ghimire A. Antibiotic resistance patterns of Staphylococcus aureus, Escherichia coli, Salmonella, Shigella and Vibrio isolated from chicken, pork, buffalo and goat meat in eastern Nepal. BMC Res Notes. 2019;12(1):766. doi: 10.1186/s13104-019-4798-7 31752992 PMC6873459

[41] Raja NS. Microbiology of diabetic foot infections in a teaching hospital in Malaysia: a retrospective study of 194 cases. J Microbiol Immunol Infect. 2007;40(1):39–44. 17332905

[42] Gaffga NH, Tauxe RV, Mintz ED. Cholera: a new homeland in Africa?. American Journal of Tropical Medicine and Hygiene. 2007;77(4):705. 17978075

[43] Hailu D, Mekonnen D, Derbie A, Mulu W, Abera B. Pathogenic bacteria profile and antimicrobial susceptibility patterns of ear infection at Bahir Dar Regional Health Research Laboratory Center, Ethiopia. Springerplus. 2016;5:466. doi: 10.1186/s40064-016-2123-7 27119070 PMC4833760

[44] Ntirenganya C, Manzi O, Muvunyi CM, Ogbuagu O. High prevalence of antimicrobial resistance among common bacterial isolates in a tertiary healthcare facility in Rwanda. Am J Trop Med Hyg. 2015;92(4):865–70. doi: 10.4269/ajtmh.14-0607 25646259 PMC4385787

[45] Kumwenda P, Adukwu EC, Tabe ES, Ujor VC, Kamudumuli PS, Ngwira M, et al. Prevalence, distribution and antimicrobial susceptibility pattern of bacterial isolates from a tertiary Hospital in Malawi. BMC Infect Dis. 2021;21(1):34. doi: 10.1186/s12879-020-05725-w 33413184 PMC7791782

[46] Nnamani KO, Nnamani CP, Iloh KK, Aghanya IN, Ushie SN, Ofiaeli OC, et al. Bacterial isolates, antibiogram and outcomes of blood culture proven sepsis in neonates at a tertiary institution in South East Nigeria: a cross-sectional study. Ther Adv Infect Dis. 2022;9:20499361221122479. doi: 10.1177/20499361221122479 36110504 PMC9468700

[47] Assefa M, Tigabu A, Belachew T, Tessema B. Bacterial profile, antimicrobial susceptibility patterns, and associated factors of community-acquired pneumonia among adult patients in Gondar, Northwest Ethiopia: A cross-sectional study. PLoS One. 2022;17(2):e0262956. doi: 10.1371/journal.pone.0262956 35104293 PMC8806065

[48] Chakraborty S. Prevalence, antibiotic susceptibility profiles and ESBL production in Klebsiella pneumoniae and Klebsiella oxytoca among hospitalized patients. Period Biol. 2016;118(1):53–8. doi: 10.18054/pb.2016.118.1.3160

[49] Gebremeskel L, Teklu T, Kasahun GG, Tuem KB. Antimicrobial resistance pattern of Klebsiella isolated from various clinical samples in Ethiopia: a systematic review and meta-analysis. BMC Infect Dis. 2023;23(1):643. doi: 10.1186/s12879-023-08633-x 37784058 PMC10544621

[50] Ameshe A, Engda T, Gizachew M. Antimicrobial Resistance Patterns, Extended-Spectrum Beta-Lactamase Production, and Associated Risk Factors of Klebsiella Species among UTI-Suspected Patients at Bahir Dar City, Northwest Ethiopia. Int J Microbiol. 2022;2022:8216545. doi: 10.1155/2022/8216545 35355926 PMC8960036

[51] Geleta D, Abebe G, Tilahun T, Gezahegn D, Workneh N, Beyene G. Phenotypic bacterial epidemiology and antimicrobial resistance profiles in neonatal sepsis at Jimma medical center, Ethiopia: Insights from prospective study. PLoS One. 2024;19(9):e0310376. doi: 10.1371/journal.pone.0310376 39283882 PMC11404823

[52] Abayneh M, HaileMariam S, Asnake M. Bacterial profile and multi-drug resistance pattern of bacterial isolates among septicemia suspected cases: a meta-analysis report in Ethiopia. Journal of Laboratory Medicine. 2021;45(3):167–78. doi: 10.1515/labmed-2020-0124

[53] Ahmed O, Omar A, Asghar A, Elhassan M. Increasing prevalence of ESBL-producing Enterobacteriaceae in Sudan community patients with UTIs. Egyptian Academic Journal of Biological Sciences, G Microbiology. 2013;5(1):17–24. doi: 10.21608/eajbsg.2013.16641

[54] Yahya Abdulla N, Abduljabbar Jaloob Aljanaby I, Hayder Hasan T, Abduljabbar Jaloob Aljanaby A. Assessment of ß-lactams and Carbapenems Antimicrobials Resistance in Klebsiella Oxytoca Isolated from Patients with Urinary Tract Infections in Najaf, Iraq. Arch Razi Inst. 2022;77(2):669–73. doi: 10.22092/ARI.2022.356957.1947 36284979 PMC9548260

[55] Yakobi SH, Nwodo UU. Prevalence of Antimicrobial Resistance in Klebsiella pneumoniae in the South African Populations: A Systematic Review and Meta-Analysis of Surveillance Studies. Microbiologyopen. 2025;14(4):e70037. doi: 10.1002/mbo3.70037 40708218 PMC12290021

[56] Sahle Z, Engidaye G, Shenkute D, Metaferia Y, Shibabaw A. High Prevalence of Multi-Drug Resistance and Extended-Spectrum Beta-Lactamase-Producing Enterobacteriaceae Among Hospitalized Patients Presumptive for Bacterial Infection at Debre Berhan Comprehensive Specialized Hospital, Ethiopia. Infect Drug Resist. 2022;15:2639–56. doi: 10.2147/IDR.S363988 35642212 PMC9148578

[57] Diriba A, et al. Prevalence, antimicrobial sensitivity patterns and associated factors of urinary tract infection among patients attending Nekemte Comprehensive Specialized Hospital, Western Ethiopia, 2024: a cross-sectional study. BMC Infectious Diseases. 2025;25(1):1–12. 40197208 10.1186/s12879-025-10788-8 PMC11977877

[58] Ibrahim Shebl R. Frequency and Antimicrobial Resistance Pattern among Bacterial Clinical Isolates Recovered from Different Specimens in Egypt. CAJPH. 2019;5(1):36. doi: 10.11648/j.cajph.20190501.16

[59] Irenge CA, Bikioli F, Mulashe PB, Kasali FM, Wimba P, Lwango A, et al. Profile of Multidrug Resistant Bacteria in Bukavu Hospitals and Antimicrobial Susceptibility to <;i>;Escherichia coli<;/i>;, <;i>;Pseudomonas aeruginosa<;/i>;, <;i>;Proteus mirabilis and Staphylococcus aureus<;/i>;. AiM. 2024;14(04):209–25. doi: 10.4236/aim.2024.144015

[60] Turay A. Etiological profiling and antimicrobial susceptibility of gram-negative uropathogens in pregnant women: A cross-sectional study at a tertiary referral center in Sierra Leone. Journal Name. 2023;60(1):1–10.

[61] Jesudason T. WHO publishes updated list of bacterial priority pathogens. Lancet Microbe. 2024;5(9):100940. doi: 10.1016/j.lanmic.2024.07.003 39079540

[62] Regassa BT, Tosisa W, Eshetu D, Beyene D, Abdeta A, Negeri AA, et al. Antimicrobial resistance profiles of bacterial isolates from clinical specimens referred to Ethiopian Public Health Institute: analysis of 5-year data. BMC Infect Dis. 2023;23(1):798. doi: 10.1186/s12879-023-08803-x 37968587 PMC10647041

[63] Kiros T, Zeleke M, Eyayu T, Workineh L, Damtie S, Andualem T, et al. Bacterial Etiology of Urinary Tract Infection and Antibiogram Profile in Children Attending Debre Tabor Comprehensive Specialized Hospital, Northwest Ethiopia. Interdiscip Perspect Infect Dis. 2023;2023:1035113. doi: 10.1155/2023/1035113 37560543 PMC10409584

[64] Amsalu A, et al. Antimicrobial resistance pattern of bacterial isolates from different clinical specimens in Southern Ethiopia: A three year retrospective study. African Journal of Bacteriology Research. 2017;9(1):1–8.

[65] Admas D, Demeke G, Adugna A, Esmael A. Bacterial etiologies, antimicrobial susceptibility pattern and associated factors among patients suspected sterile body site infections at Debre Markos Comprehensive Specialized Hospital, Northwest Ethiopia. Front Med (Lausanne). 2024;11:1260841. doi: 10.3389/fmed.2024.1260841 38774397 PMC11106364

[66] Zenebe Y, et al. Bacterial profile and antimicrobial susceptibility pattern of neonatal sepsis in Felege-Hiwot Referral Hospital, Bahir Dar, northwest Ethiopia: A cross-sectional study design. Ethiopian Journal of Health Development. 2021;35(1).

[67] Belete Y, et al. Bacterial profile and antibiotic susceptibility pattern of urinary tract infection among children attending Felege Hiwot Referral Hospital, Bahir Dar, Northwest Ethiopia. Infection and Drug Resistance. 2019;:3575–83. 31819542 10.2147/IDR.S217574 PMC6874112

[68] Godebo G, Kibru G, Tassew H. Multidrug-resistant bacterial isolates in infected wounds at Jimma University Specialized Hospital, Ethiopia. Ann Clin Microbiol Antimicrob. 2013;12:17. doi: 10.1186/1476-0711-12-17 23879886 PMC3724577

[69] Gill JS, Arora S, Khanna SP, Kumar KH. Prevalence of Multidrug-resistant, Extensively Drug-resistant, and Pandrug-resistant Pseudomonas aeruginosa from a Tertiary Level Intensive Care Unit. J Glob Infect Dis. 2016;8(4):155–9. doi: 10.4103/0974-777X.192962 27942195 PMC5126754

[70] Pattnaik D, Panda SS, Singh N, Sahoo S, Mohapatra I, Jena J. Multidrug resistant, extensively drug resistant and pan drug resistant gram negative bacteria at a tertiary care centre in Bhubaneswar. Int J Community Med Public Health. 2019;6(2):567. doi: 10.18203/2394-6040.ijcmph20190170

[71] Wang M, Wei H, Zhao Y, Shang L, Di L, Lyu C, et al. Analysis of multidrug-resistant bacteria in 3223 patients with hospital-acquired infections (HAI) from a tertiary general hospital in China. Bosn J Basic Med Sci. 2019;19(1):86–93. doi: 10.17305/bjbms.2018.3826 30579325 PMC6387671

[72] Manyahi J, Kibwana U, Mgimba E, Majigo M. Multi-drug resistant bacteria predict mortality in bloodstream infection in a tertiary setting in Tanzania. PLoS One. 2020;15(3):e0220424. doi: 10.1371/journal.pone.0220424 32130227 PMC7055912

[73] Leski TA, Taitt CR, Bangura U, Stockelman MG, Ansumana R, Cooper WH 3rd, et al. High prevalence of multidrug resistant Enterobacteriaceae isolated from outpatient urine samples but not the hospital environment in Bo, Sierra Leone. BMC Infect Dis. 2016;16:167. 10.1186/s12879-016-1495-1 27090787 PMC4836052 27090787

[74] Agyepong N, Govinden U, Owusu-Ofori A, Essack SY. Multidrug-resistant gram-negative bacterial infections in a teaching hospital in Ghana. Antimicrob Resist Infect Control. 2018;7:37. doi: 10.1186/s13756-018-0324-2 29541448 PMC5845144

[75] Sleem AS, et al. Prevalence of multidrug-resistant bacteria isolated from patients with burn infection. Menoufia Medical Journal. 2015;28(3):677–84.

[76] Basak S, Singh P, Rajurkar M. Multidrug Resistant and Extensively Drug Resistant Bacteria: A Study. J Pathog. 2016;2016:4065603. doi: 10.1155/2016/4065603 26942013 PMC4749793

[77] Awasthi TR, Pant ND, Dahal PR. Prevalence of multidrug resistant bacteria in causing community acquired urinary tract infection among the patients attending outpatient department of Seti Zonal Hospital, Dhangadi, Nepal. Nepal J Biotechnol. 2015;3(1):55–9. doi: 10.3126/njb.v3i1.14232

[78] Lim CJ, Cheng AC, Kennon J, Spelman D, Hale D, Melican G, et al. Prevalence of multidrug-resistant organisms and risk factors for carriage in long-term care facilities: a nested case-control study. J Antimicrob Chemother. 2014;69(7):1972–80. doi: 10.1093/jac/dku077 24710025

[79] Adrizain R, Suryaningrat F, Alam A, Setiabudi D. Incidence of multidrug-resistant, extensively drug-resistant and pan-drug-resistant bacteria in children hospitalized at Dr. Hasan Sadikin general hospital Bandung Indonesia. IOP Conf Ser: Earth Environ Sci. 2018;125:012077. doi: 10.1088/1755-1315/125/1/012077

[80] Aliyu S, Smaldone A, Larson E. Prevalence of multidrug-resistant gram-negative bacteria among nursing home residents: A systematic review and meta-analysis. Am J Infect Control. 2017;45(5):512–8. doi: 10.1016/j.ajic.2017.01.022 28456321

[81] Buke C, Armand-Lefevre L, Lolom I, Guerinot W, Deblangy C, Ruimy R, et al. Epidemiology of multidrug-resistant bacteria in patients with long hospital stays. Infect Control Hosp Epidemiol. 2007;28(11):1255–60. doi: 10.1086/522678 17926276

[82] Jonas N. Prevalence and antimicrobial susceptibility pattern of gram-negative bacteria contaminating the hands of patients’ visitors at regional referral hospitals in Dar-es-Salaam: A hospital based cross sectional study. 2024. 10.1371/journal.pone.0320700 PMC11940574 40138326

[83] Alemayehu T. Prevalence of multidrug-resistant bacteria in Ethiopia: a systematic review and meta-analysis. J Glob Antimicrob Resist. 2021;26:133–9. doi: 10.1016/j.jgar.2021.05.017 34129993

[84] Demsie DG, Addisu ZD, Tefera BB, Gebrie D, Tsegay EW, Yehualaw A, et al. Knowledge, and attitude as determinants of healthcare professionals’ self-medication practice to antibacterials in Tertiary Care hospitals, North West Ethiopia. Sci Rep. 2025;15(1):5241. doi: 10.1038/s41598-025-88979-1 39939667 PMC11822107

