# Supplementary material for: Antimicrobial resistance profiles of bacteria from clinical specimens at Amhara Public Health Institute, Bahir Dar, Ethiopia: A retrospective study
Source: PLoS One. 2025 Dec 5;20(12):e0337332. doi: 10.1371/journal.pone.0337332 (PMC12680254; doi:10.1371/journal.pone.0337332)
Supplement: Attachment — Submitted filename: Response to Reviewers.docx [file pone.0337332.s005.docx]

PONE-D-25-08930

Title: Antimicrobial resistance profiles of bacterial from clinical specimens in Amhara National Regional State Public Health Institute, Bahir Dar, Ethiopia: a retrospective study

**Dear Academic Editor PLOS ONE and Reviewers,**

We would like to begin by thanking the editor and reviewers for their time spent revewing this manuscript and for their many helpful comments. We have addressed all the suggestions in our current version and we believe the manuscript has been improved. In this letter, our responses are written in purple. We have included a marked-up copy (word) of the manuscript that highlights changes made to the original version are upload as a separate file labeled 'Revised Manuscript with Track Changes and unmarked version of our revised paper without tracked changes are upload as a separate file labeled 'Manuscript'.

**Editor Comments#1**

**Please include the following items when submitting your revised manuscript:**

1. **A rebuttal letter that responds to each point raised by the academic editor and reviewer(s). You should upload this letter as a separate file labeled 'Response to Reviewers'.**

**Response:** We resubmit with a rebuttal letter that includes list of responses to the editors' and reviewers' comments.

2. **A marked-up copy of your manuscript that highlights changes made to the original version. You should upload this as a separate file labeled 'Revised Manuscript with Track Changes'.**

**Response:** As you suggested, we have uploaded the manuscript markup after making the necessary track changes.

3. **An unmarked version of your revised paper without tracked changes. You should upload this as a separate file labeled 'Manuscript'.**

**Response:** We also upload an unmarked version of our revised manuscript without tracked changes labled as “Manuscript”

4. **If you would like to make changes to your financial disclosure, please include your updated statement in your cover letter.**

**Response:**We had neither funding support nor funding to report, thus we wouldn't alter the financial disclosure.

5. **Guidelines for resubmitting your figure files are available below the reviewer comments at the end of this letter.**

**Response:** We adapted figure files to meet the style requirements

**Editor Comment # 2**

6. **Please ensure that your manuscript meets PLOS ONE's style requirements, including those for file naming.The PLOS ONE style templates can be found at** [**https://journals.plos.org/plosone/s/file?id=wjVg/PLOSOne_formatting_sample_main_body.pdf**](https://journals.plos.org/plosone/s/file?id=wjVg/PLOSOne_formatting_sample_main_body.pdf) **and** [**https://journals.plos.org/plosone/s/file?id=ba62/PLOSOne_formatting_sample_title_authors_affiliations.pdf**](https://track.editorialmanager.com/CL0/https:%2F%2Fjournals.plos.org%2Fplosone%2Fs%2Ffile%3Fid=ba62%2FPLOSOne_formatting_sample_title_authors_affiliations.pdf/1/010f0196e7983296-78ebc0fa-0a4e-4be3-8c9e-5f6ff2d13600-000000/xsXqymp4pxw1Mr2ejEPKOnmaMs49KsYoZN-DMrtV3Y8=211)

**Response:** We adapted the manuscript to meet the style requirements.

7. **We require the following information in order to proceed with your submission: As you are reporting a retrospective study of medical records or archived samples, please ensure that you have discussed whether all data were fully anonymized before you accessed them and/or whether the IRB or ethics committee waived the requirement for informed consent. If patients provided informed written consent to have data from their medical records used in research, please include this information in the Methods section.**

**Response:** A waiver of consent is admissible if it is impossible or very difficult to contact each patient and obtain his or her specific consent for the retrospective research.

8. **We note that you have indicated that there are restrictions to data sharing for this study. PLOS only allows data to be available upon request if there are legal or ethical restrictions on sharing data publicly. For more information on unacceptable data access restrictions, please see** [**http://journals.plos.org/plosone/s/data-availability#loc-unacceptable-data-access-restrictions**](http://journals.plos.org/plosone/s/data-availability#loc-unacceptable-data-access-restrictions)**.**

**Before we proceed with your manuscript, please address the following prompts:**

a) **If there are ethical or legal restrictions on sharing a de-identified data set, please explain them in detail (e.g., data contain potentially identifying or sensitive patient information, data are owned by a third-party organization, etc.) and who has imposed them (e.g., a Research Ethics Committee or Institutional Review Board, etc.). Please also provide contact information for a data access committee, ethics committee, or other institutional body to which data requests may be sent.**

**Response:** We will make the necessary changes. The corresponding author can provide the datasets used and/or analyzed in the current work upon reasonable request.

**b) If there are no restrictions, please upload the minimal anonymized data set necessary to replicate your study findings to a stable, public repository and provide us with the relevant URLs, DOIs, or accession numbers. For a list of recommended repositories, please see** [**https://journals.plos.org/plosone/s/recommended-repositories**](https://journals.plos.org/plosone/s/recommended-repositories)**. You also have the option of uploading the data as Supporting Information files, but we would recommend depositing data directly to a data repository if possible.We will update your Data Availability statement on your behalf to reflect the information you provide**.

**Response:** We uploading the data as supporting Information files

9. **Your ethics statement should only appear in the Methods section of your manuscript. If your ethics statement is written in any section besides the Methods, please move it to the Methods section and delete it from any other section. Please ensure that your ethics statement is included in your manuscript, as the ethics statement entered into the online submission form will not be published alongside your manuscript.**

**Response:**We remove the ethical statement from all other sections and relocate it to the Methods section.

10. We are unable to open your Supporting Information file “Suporting information.rar”. Please kindly revise as necessary and re-upload.

**Response:** We have fixed the problem and re-uploaded the supporting documentation file.

**Additional Editor Comments:**

11. **The paper generally requires intensive revision. That is, it requires a through edition, revision and proofreading in terms of typography, punctuation and grammatically.**

**Response:**Before we submitted the manuscript to PLOS ONE, we would have it edited, revised, and checked by a professional language editor for typography, punctuation, and grammar.

12. The background of the abstract was no explanatory.

Response: We make is explanatory

13. The abstract part of study period should be one and remove the other.

**Response:** we remove the other

14. Consistent use of the words/phrases such as drug, antimicrobial or antibiotics is necessary.

**Response:** We create it.Regular usage of the terms

15. It is better the author use standard form of Gram-positive or Gram-negative

**Response:** We included consistence usage of the terms

16. In the abstract and result, the absolute number (numerator and denominator) is needed together with the percentage. For example, A/B (C%).

**Response: We included the absolute number (numerator and denominator)**

17. Write the name of the bacteria at its first appearance, no abbreviation is needed; then for the second appearance write the abbreviated form and continue. Here, please rewrite the abstract part.

**Response:** We correct it

18. **Revise also the conclusion based on the findings**

**Response:** We revise the conclusion in light of our findings.

19. The introduction should be written in terms of burden of bacteria, rationality and objectives of the study.

**Response:** We update the background information to reflect our topics

20. The discussion seems like result and requires revision.

**Response:** We will amend it

21. The discussion should be supported by reasons for variation or similarity, not merely comparison.

**Response:** We will amend it

22. Figures should be uploaded in separate file as ‘TIF’ version

**Response:** We adapted figure files to meet the style requirements

23. Follow properly the manuscript writing protocol for PLoS one.

**Response:** We adapted the manuscript to meet the style requirements

**Reviewer Comments**

24. **Reviewer #1: The author can see the detailed from the attached pdf
    The authors should clearly explain what is known and unknown about the study?**

**Response:** We have clearly explain what is known and unknown about the study

25. **Comment 1:** In the abstract line 63 why the overall antimicrobial resistance profiles seen in your study are of a big concern? What is your reference to say big, medium and low?

**Response: we have amend it**

26. **Comment 2:** On the introduction part line 75-78 stated without evidence so the author should cite the source

**Response: we have put reference**

27. **Comment** 3: The authors should clearly explain what is known and unknown about the study?

**Response: We have clearly explain what is known and unknown about the study**

28. **Comment** 4: On the methodology part you have you used cross sectional study design but I didn’t have seen the elaboration about sampling. Does your study is survey type?

**Response: we have amend it**

29. **Comment** 5: On Operational definitions line 212 to 216 if you think the terms are already known by readers leave it no need of define. But if it is obligated the authors should cite the appropriate reference

**Response: we have corrected it**

30. **Comment** 6: Result part table 1 how can you categorized age please put the source you have used

Response: The age groups 0-4, 5-14, 15-64, and 65+ are commonly used in demographic analysis and population statistics to categorize individuals. Here below are some source used as reference.

1.Bou-Antoun S, Falola A, Fountain H, Squire H, Brown CS, Hopkins S, Gerver SM, Demirjian A. Antimicrobial Consumption in England, 2017 to 2021. InMedical Sciences Forum 2023 Jan 18 (Vol. 15, No. 1, p. 1). MDPI.

2. Cedrone F, Montagna V, Del Duca L, Camplone L, Mazzocca R, Carfagnini F, Fortunato V, Di Martino G. The burden of Streptococcus pneumoniae-related admissions and in-hospital mortality: a retrospective observational study between the years 2015 and 2022 from a southern Italian province. Vaccines. 2023 Aug 4;11(8):1324.

31. **Comment** 7: On discussion part the authors compare different findings in different study area but also, they should explain the rationality for each discrepancy behind each finding.

**Response: we have explain the rationality for each discrepancy behind each finding**

32. **Comment** 8: Have you got complete information for your study? I think in secondary retrospective study especially in document review missing data is one challenge so how can you solve

**Response:** Yes, missing values are a common problem in secondary retrospective study especially, but our dataset contains missing values for the dependent and independent variable were very small and exclude those records.

33. **On the methodology part you have you used cross sectional study design but I didn’t have seen the elaboration about sampling. Does your study is survey type?**

**Response: our study were retrospective, so we have amend it.**

34. **On Operational definitions line 212 to 216 if you think the terms are already known by readers leave it no need of define. But if it is obligated the authors should cite the appropriate reference**

**Response: we amend it**

**Reviewer #2:**

**Thank you for the opportunity to review the manuscript entitled "Antimicrobial resistance profiles of bacterial from clinical specimens in Amhara National Regional State Public Health Institute, Bahir Dar, Ethiopia: a retrospective study". This manuscript addresses the critical issue of antimicrobial resistance (AMR) in a high-burden setting, providing valuable surveillance data from a regional reference laboratory in Ethiopia. The topic is highly relevant globally, particularly for low- and middle-income countries (LMICs) where AMR surveillance is often limited. The retrospective study design and data from clinical specimens over years offer insight into local resistance trends that can inform public health interventions and antimicrobial stewardship. However, while the study has merit, several aspects require major revision before it can be considered for publication.**

**Response:** I appreciate your kind feedback very much.

35. **Comment 1 (General comment on the manuscript): The manuscript requires extensive language editing. Numerous grammatical issues and awkward phrasing obscure key points (e.g., “big concern” should be “serious concern”). The narrative lacks clarity in places, particularly in the results and discussion sections. Consider professional English editing.**

Response: We agree with the reviewer’s comment. Accordingly, We went throughout the entire manuscript to eliminate language, grammatical and awkward phrasing issues. These issues have been curiously amended.

36. **Comment-2 (Title): Better if edited like as: Antibiotic Resistance Profiles of Bacteria Isolated from Clinical Specimens at Amhara National Regional State Public Health Institute, Bahir Dar, Ethiopia: A Retrospective Study. Antibiotic resistance" replaces "antimicrobial resistance" to specifically target bacterial pathogens and Bacteria isolated from clinical specimens" improves clarity and grammatical flow.**

**Response: we correcte it**

37. **Comment 3: The abstract is overly detailed for a structured abstract, (should be within 250-300 words).**

**Response:** To make the abstract understandable and illustrative, we had to reduce its size.

38. **Comment 4: Consider shortening results to key findings with precise percentages for major resistant pathogens. Ensure consistent formatting of species names (e.g., Escherichia coli, Staphylococcus aureus) in italics throughout. Do not use abbreviation and also both in the abstract.**

**Response:**According to your feedback, we make sure that species names are formatted consistently and concentrate on key findings with precise percentages for major resistant pathogens. we avoided using abbreviations.

39. **Comment 5: In line number 36: “The drug susceptibility test was performed using the Kirby-Bauer disc diffusion method on Muller-Hinton agar” who was performed the AST? Please make it clear or omit. And also use the specific term like antibiotic rather drug.**

**Response: we amend it**

40. **Comment 6: Reduce redundancy of information, like study period, you have mentioned in both background and methods.**

**Response:** we appreciate your comments . We immediately make the necessary changes.

41. **Comment 7: In the results section (lines 38–62), please include only the key findings with precise percentages for major resistant bacterial pathogens, and omit the fungal findings, as your study focuses on bacteria, not all microorganisms.**

Response:According to your feedback, we make sure that species names are formatted consistently and concentrate on key findings with precise percentages for major resistant pathogens. we avoided using abbreviations.

42. **Comment 8: The conclusion effectively highlights the public health implications of the findings. However, consider strengthening the impact by specifying the most concerning resistant pathogens identified in your study. This would better tie the conclusion to your results and emphasize the urgency of surveillance and stewardship efforts.**

**Response:** We are also grateful for the suggestion and thank you. We amend it based on your suggestion.

43. **Comment 9: Rewrite “Material and Methods” like as Methods and Materials**

**Response:** Thank you for the suggestion. We amend it now

44. **Comment 10: Break specifically “Study design, period and area” and maintain consistency**

**Response: Thank you and we also appreciate your suggestion. We amend it**

45. **Comment 11: There is a lack of detail regarding quality control measures and breakpoint interpretation standards (e.g., CLSI 2021 or 2022?). Ensure that versions are cited and methodological adherence is clear.**

**Response:** We are grateful for the suggestion and thank you. We try re-write in detail regarding to quality control measures and breakpoint interpretation standards.

46. **Comment 12: Study population (line number132-136) “….a patients who visited APHI Medical Microbiology Reference Laboratory and had complaints of any infection suspected of microbial infections during the study period”. As you know your study was retrospective so make it clear to reduce confusion with prospective aspects.**

**Response:** Thank you for the suggestion. We amend it now

47. **Comment 13: Consider clarifying inclusion/exclusion criteria, e.g., how incomplete records were managed and whether duplicates were removed.**

**Response:**Thank you, and we value your suggestion as well. We are making the inclusion/exclusion criteria more clear.

48. **Comment 14: Line number 146 “Specimen collection, processing, and bacterial identifications” since your study was retrospective, thus, it is better to elaborate more about the recorded data, the way of collection and maintaining of the quality of data rather than stating about diagnostic process.**

**Response:** Thank you, and we value your suggestion as well. We also elaborate more about the recorded data, the way of collection and maintaining of the quality of data

49. **Comment 15: Please recheck and rewrite/rephrase methods and materials section by considering your source of data (line number 118-204)**

**Response: Thank you, we include addtionals on the methods and materials**

50. **Comment 16: The results are comprehensive but not well-structured. Tables are very large and difficult to interpret.**

**Response:** Thank you; we have added more details on the methods and materials

51. **Comment 17: Consider focusing on the most clinically relevant pathogens (e.g., E. coli, K. pneumoniae, S. aureus) in the main text and shifting exhaustive antibiograms to supplementary material.**

**Response:** Thanks for the remark. We are concentrating on the the most clinically relevant pathogens, and we have put the antibiograms to supplementary material.

52. **Comment 18: Define key terms such as "MDR" clearly within the results, and consider reporting "XDR" and "PDR" if applicable.**

**Response:** I appreciate your comment. However, I define important words like MDR in the section on methods and materials.

**Discussion section:**

53. **Comment 19: The discussion is lengthy, repetitive, and lacks a clear focus on clinical and policy implications.**

**Response:** We appreciate your feedback and will consider the reviewer's concerns.

54. **Comment 20: There is limited critical reflection on factors contributing to resistance in the local context (e.g., prescribing practices, diagnostics access, stewardship programs).**

**Response:** We appreciate your suggestion as well, but the limited availability of clinical virabiles in the microbiological log book prevented us from evaluating the factors contributing to resistance.

55. **Comment 21: Compare findings with WHO GLASS data and other sub-Saharan Africa studies more systematically.**

**Response: We also appreciate your comment, and we compare our results with research conducted in sub-Saharan Africa.**

**Ethical clearance section:**

56. **Comment 22: Ethical approval is adequately described. However, the data availability statement must align with PLOS ONE policies; simply stating "data within the manuscript" may not be sufficient.**

**Response: Thank you for your comment. We modified the data availability statement accordingly.**

Sincerely,

Michael Getie ( Corresponding author)
