# Supplementary material for: Antimicrobial resistance profiles of bacteria from clinical specimens at Amhara Public Health Institute, Bahir Dar, Ethiopia: A retrospective study
Source: PLoS One. 2025 Dec 5;20(12):e0337332. doi: 10.1371/journal.pone.0337332 (PMC12680254; doi:10.1371/journal.pone.0337332)
Supplement: Attachment — Submitted filename: Article Review Report.docx [file pone.0337332.s004.docx]

**Article Review Report**

**Title:** Antimicrobial Resistance Profiles of Bacteria from Clinical Specimens at Amhara National Regional State Public Health Institute, Bahir Dar, Ethiopia: A Retrospective Study

- longer, reduce the length for more brevity

**Abstract**

- Line 28: Replace "antimicrobial resistance pattern" with "antimicrobial resistance profile" for consistency with the title.
- Line 32: Specify the age range of patients included in the laboratory results
- Punctuation: Add a colon (:) after "Background" and "Methods."
- Line 31: It is generally preferred to use "July 1, 2022, to December 31, 2024" instead of "1/7/2022 to 31/12/2024.
- Line 36: If data were collected, please indicate the tools used; if the data were extracted rather than collected, please clarify that.
- Line 37: In "descriptive statistics," it would improve clarity to specify the types of analyses conducted (e.g., frequency distributions, chi-square tests).
- Line 40: add denominator for 41.4% (95% CI: 39; 44) and indicate in your mother document the section that show stool (263; 54.6%), urine (98; 20.3%), wound (62; 12.9%), blood (47; 9.8%).
- In line 42-48: if you are reporting bacteria just use the total bacterial isolates which is 478

**Methods and Materials**

rewrite "Methods and Material" as: Methods and Materials

**Study area**

- Include details about the type of culturing and the data handling and storing system to ensure consistency with WHONET,
- In line 120-21, data records of two and a half years of data from 1/7/2022 to 31/12/2024; both times operationally similar. Use one of them.
- In line 122-4, the objective is already stated under introduction, no need to repeat
- In line 131-4, ….. The study utilized an entire sampling technique that included all available microbiology culture records of patients of all age groups suspected of having microbial infections during study period. What is that entire sampling technique?

**Inclusion and exclusion criteria**

- In line 138, Patient seating indicates?

**Bacterial identification**

- In line 151, indicate the year for CLSI

Data source and access

No comment

Specimen and Data quality control

No comment

**Data analysis**

Include my comment from abstract section

Ethics approval

In line 125-6: This manuscript does not report on or involve the use of any animal or human data or tissue. Be sure of this?

**Results**

**Line 260**: Please remove "Table 1" from the sentence "most frequently culture-processed specimens.

**Culture Results in Table 2**: Refine the percentages by calculating them based on the total sample denominator. Additionally, since the title focuses on antimicrobial resistance profiles of bacteria, consider excluding **Candida albicans** from the analysis or modifying the title accordingly.

Please provide a rationale for any variables that were not analyzed.

**Discussion**

- Lines 381-382: The study reported an overall bacterial isolation frequency of 41.4% (482 isolates) from clinical specimens. However, this total includes 4 fungal isolates, which are not classified as bacterial isolates and need your action
- In line 390: Better to move this explanation after line 390 ( The most possible explanation could be due to the difference in culture identification technique in the study population, the study design, geographical location, etiological agents, and infection prevention and control policies between regions and countries [2, 13, and 24]).
- Reference at end of line 408 and 466

**Reference**

- Check for correctness of reference for number 4(format), 7(format and version), 24 (format) and 46 (format)
