# Supplementary material for: Antimicrobial resistance profiles of bacteria from clinical specimens at Amhara Public Health Institute, Bahir Dar, Ethiopia: A retrospective study
Source: PLoS One. 2025 Dec 5;20(12):e0337332. doi: 10.1371/journal.pone.0337332 (PMC12680254; doi:10.1371/journal.pone.0337332)
Supplement: Attachment — Submitted filename: Response_to_Reviewers_auresp_2.docx [file pone.0337332.s006.docx]

Mr.Tebelay Dilnessa,

Academic Editor
PLOS ONE
24/8/2025
Dear Mr. Tebelay,

We are grateful for the opportunity to submit a revised draft of our manuscript entitled, " Antimicrobial Resistance Profiles of Bacteria from Clinical Specimens at Amhara Public Health Institute, Bahir Dar, Ethiopia: A Retrospective Study" to PLOS ONE. We also appreciate the time and effort you and each of the reviewers have dedicated to providing insightful feedback on ways to strengthen our paper. Thus, it is with great pleasure that we resubmit our article for further consideration. We have incorporated changes that reflect the detailed suggestions you have graciously provided. We also hope that our edits and the responses we provide below satisfactorily address all the issues and concerns you and the reviewers have noted.
To facilitate your review of our revisions, the following is a point-by-point response to the questions and comments delivered in your letter dated 24/8/2025.
 **Editor Comments:**

1) The paper was improved, still it requires revision. I would like to recommend you that you have to revise it properly.

- Response: We also thank you for this, we have properly revise the manuscript.

2) Line 104: Materials and methods

- Response: We also thank you for this, we have corrected this part.

3) Lines 60 &61: It is better the author use standard form of Gram-positive or Gram-negative (previous comment).

- Response: Thank you for providing these insights. We correct it

4) Lines 106 and 107: The study was conducted at APHI, Bahir Dar city. Which is 565 km away from Addis Ababa (the capital of Ethiopia) a………Please revise it, based on the context, there is no sentence that starts by using ‘Which’.

- Response: I also thank you for this, we have corrected it.

5) Write separately ‘study population’ and ‘sampling technique’.

- Response: I also thank you for this, we have corrected this part

6) Line 144: Bacterial isolation and identification

- Response: Thank you for your suggestion. We have included it.

7) Line 206: Data quality control

- Response: Thank you for your suggestion. We have corrected it.

8) I recommend a separate heading as ‘Sociodemographic characteristics of study participants’ and ‘Magnitudes of bacteria’.

- Response: we accepted your comment and we have put it separately.

9) Rewrite from line 251 to 267. A sentence cannot start just by saying ‘Table 1:……’

- Response: I also thank you for this, which was clerical error we have corrected.

10) Line 270: What does it mean ‘Table 2’?

Response: I also thank you for this. We have corrected these clerical errors.

11) In table 2, you considered ‘***Candida albicans’*** as bacteria. Is that correct? Please revise.

- Response: We accepted your comment and we corrected it

12) In the abstract and result, the absolute number (numerator and denominator) is needed together with the percentage. For example, A/B (C%) (previous comment).

- Response: We have considered your comments, and we have included both numerator and denominator together with the percentage.

13) Line 311 and 326: Gram-negative

- Response: Thank you for providing these insights. We have corrected this part.

14) Line 334, 346, etc: Gram-positive

- Response: Thank you for providing these insights. We have made a correction.

**Reviewer #3:**

1. longer, reduce the length for more brevity

- Response: Thank you for providing these insights. We have made a correction to reduce title.

**Abstract**

2. Line 28: Replace "antimicrobial resistance pattern" with "antimicrobial resistance profile" for consistency with the title.

- Response: I also thank you for this, we have corrected this part

3. Line 32: Specify the age range of patients included in the laboratory results

- Response: We accepted your comment and included the age range of patients in the abstract and results section.

4. Punctuation: Add a colon (:) after "Background" and "Methods."

- Response: I also thank you for this, we have corrected this part.

5. Line 31: It is generally preferred to use "July 1, 2022, to December 31, 2024" instead of "1/7/2022 to 31/12/2024.

- Response: I also thank you for this, we have corrected this part.

6. Line 36: If data were collected, please indicate the tools used; if the data were extracted rather than collected, please clarify that.

- Response: We agree with you and we have extracted WHO NET 2024 database for the study, and we explained the how data extracted for this study

7. Line 37: In "descriptive statistics," it would improve clarity to specify the types of analyses conducted (e.g., frequency distributions, chi-square tests).

- Response: Thank you for your suggestion. We have included types of analyses conducted.

8. Line 40: add denominator for 41.4% (95% CI: 39; 44) and indicate in your mother document the section that show stool (263; 54.6%), urine (98; 20.3%), wound (62; 12.9%), blood (47; 9.8%).

- Response: Thank you for your suggestion. We have corrected it.

9. In line 42-48: if you are reporting bacteria just use the total bacterial isolates which is 478

- Response: We agree with you and we have revised and re-write the result section based on your suggestion.

**Methods and Materials**

10. rewrite "Methods and Material" as: Methods and Materials

- Response: We also thank you for this, we have corrected this part.

**Study area**

11. Include details about the type of culturing and the data handling and storing system to ensure consistency with WHONET,

	Response: We also thank you for this, we mentioned details about the type of culturing and the data handling and storing.

12. In line 120-21, data records of two and a half years of data from 1/7/2022 to 31/12/2024; both times operationally similar. Use one of them.

- Response: I also thank you for this, we have corrected it.

13. In line 122-4, the objective is already stated under introduction, no need to repeat

- Response: We also thank you for this, we have avoid redundancy.

14. In line 131-4, ….. The study utilized an entire sampling technique that included all available microbiology culture records of patients of all age groups suspected of having microbial infections during the study period. What is that entire sampling technique?

- Response: We also thank you for this. We utilize the entire sampling technique to describe a comprehensive sampling approach that includes complete available bacteriological culture records of patients on WHONET 2024 database during the study period.

**Inclusion and exclusion criteria**

15. In line 138, Patient seating indicates?

- Response: Thank you for your suggestion. We have rephrased “Patient seating” as hospital units refer to specific divisions within a hospital, including emergency departments (EDs) and outpatient departments (OPDs), inpatient ward that provide distinct healthcare services and are sampled separately for data collection.

**Bacterial identification**

16. In line 151, indicate the year for CLSI

- Response: Thank you for providing these insights. We included year for CLSI.

**Data analysis**

17. Include my comment from abstract section

- Response: Thank you for your suggestion. We have corrected based on your pervious comments.

Ethics approval

18. In line 125-6: This manuscript does not report on or involve the use of any animal or human data or tissue. Be sure of this?

- Response: Thank you for providing these insights. We amend it.

**Results**

19. **Line 260**: Please remove "Table 1" from the sentence "most frequently culture-processed specimens.

- Response: I also thank you for this, which was clerical error we have corrected this it.

20. **Culture Results in Table 2**: Refine the percentages by calculating them based on the total sample denominator. Additionally, since the title focuses on antimicrobial resistance profiles of bacteria, consider excluding **Candida albicans** from the analysis or modifying the title accordingly.

- Response: we accepted your comment and we corrected it

21. Please provide a rationale for any variables that were not analyzed.

- Response: We thank you for your nice comments. As we know, antimicrobial agents were selected for testing and reporting based on the CLSI M100 performance standard for antimicrobial susceptibility testing. Recommendations for each organism group include agents of proven test efficacy that show acceptable in vitro test susceptibility. But bacterial isolates may appear active in vitro but are not effective clinically and should not be analyzed and reported as susceptible.

**Discussion**

22. Lines 381-382: The study reported an overall bacterial isolation frequency of 41.4% (482 isolates) from clinical specimens. However, this total includes 4 fungal isolates, which are not classified as bacterial isolates and need your action

- Response: we accepted your comment and we corrected it

23. In line 390: Better to move this explanation after line 390 ( The most possible explanation could be due to the difference in culture identification technique in the study population, the study design, geographical location, etiological agents, and infection prevention and control policies between regions and countries [2, 13, and 24]).

- Response: We agree with you and corrected it

24. Reference at end of line 408 and 466

- Response: We thank you for this comments, we have cited reference for paragraph line 408 and 466.

**Reference**

25. Check for correctness of reference for number 4(format), 7(format and version), 24 (format) and 46 (format)

- Response: Thank you for providing these insights. We have checked for correctness of references.
