# Supplementary material for: Antimicrobial resistance profiles of bacteria from clinical specimens at Amhara Public Health Institute, Bahir Dar, Ethiopia: A retrospective study
Source: PLoS One. 2025 Dec 5;20(12):e0337332. doi: 10.1371/journal.pone.0337332 (PMC12680254; doi:10.1371/journal.pone.0337332)
Supplement: Attachment — Submitted filename: reviewer comment ploseeone.pdf [file pone.0337332.s002.pdf]

First, I would like thanks the authors conducted a study such a very important topic which is the current medical treatment challenges next, I thanks the editor providing the privilege to review this paper.

1. In the abstract line 63 why the overall antimicrobial resistance profiles seen in your study are of a big concern? What is your reference to say big, medium and low?
2. On the introduction part line 75-78 stated without evidence so the author should cite the source
3. The authors should clearly explain what is known and unknown about the study?
4. On the methodology part you have you used cross sectional study design but I didn’t have seen the elaboration about sampling. Does your study is survey type?
5. On Operational definitions line 212 to 216 if you think the terms are already known by readers leave it no need of define. But if it is obligated the authors should cite the appropriate reference
6. Result part table 1 how can you categorized age please put the source you have used
7. On discussion part the authors compare different findings in different study area but also, they should explain the rationality for each discrepancy behind each finding.
8. Have you got complete information for your study? I think in secondary retrospective study especially in document review missing data is one challenge so how can you solve ?
